# Percutaneous screw osteosynthesis for the treatment of intra-articular displaced calcaneus fractures

**DOI:** 10.1007/s00068-026-03098-4

**Published:** 2026-04-21

**Authors:** Leah Wilmsen, Anne Neubert, Adrian Deichsel, Denise Schulz, Stefanie Hoelscher-Doht, Joachim Windolf, Andrea Icks, Simon Thelen

**Affiliations:** 1 General medicine practice, Langeoog, Germany; 2 Department of Orthopaedics and Traumatology, Medical Faculty and University Hospital Duesseldorf, Heinrich-Heine-University Duesseldorf, Duesseldorf, Germany; 3 TraumaEvidence @ German Trauma Society, Berlin, Germany; 4 Department of Trauma, Hand and Reconstructive Surgery, University Hospital Muenster, Muenster, Germany; 5 Department of Trauma, Hand, Plastic and Reconstructive Surgery, University Hospital Wuerzburg, Wuerzburg, Germany; 6 University Hospital RWTH Aachen, Aachen, Germany; 7 Institute for Health Services Research and Health Economics, Centre for Health and Society (chs), Medical Faculty, University Hospital Duesseldorf, Heinrich-Heine- University Duesseldorf, Duesseldorf, Germany; 8 Heinrich-Heine-University Duesseldorf, Duesseldorf, Germany

**Keywords:** Calcaneus fracture, Percutaneous screw osteosynthesis, ORIF, Systematic review

## Abstract

**Purpose:**

This systematic review investigates how percutaneous screw osteosynthesis for the treatment of intra-articular displaced calcaneus fractures differs from open reduction and internal fixation (ORIF).

**Methods:**

Randomized controlled trials and controlled clinical trials investigating adults with closed intra-articular displaced calcaneus fracture (Sanders type II - IV) treated with percutaneous screw osteosynthesis or ORIF were included. Severe vascular and neurological diseases or polytrauma patients were excluded. On January 29, 2024, five databases and two trial registries were searched. The Risk of Bias Tool of Cochrane was used. The protocol was registered on PROSPERO (CRD42021244695).

**Results:**

Five studies with 654 participants (682 calcaneus fractures) were included. The risk of bias was moderate to high. None of the single studies found a significant difference regarding severe complications. However, meta-analysis revealed a lower risk for the percutaneous screw osteosynthesis group compared to ORIF (relative risk (RR) 0.40 (95% Confidence Interval [0.17; 0.92]; I² = 0%; *p* = 0.03). One study showed less pain in the percutaneous screw osteosynthesis group up to 4 weeks postoperatively. No study assessed health-related quality of life.

**Conclusion:**

Percutaneous screw osteosynthesis in intra-articular displaced calcaneus fractures might be beneficial to ORIF in terms of minor and severe complications, and pain at four weeks. Percutaneous screw osteosynthesis could be an additional, less invasive treatment option, depending on the complexity of the injury. However, studies with larger sample sizes are needed to confirm these results and explore long-term complications.

**Supplementary Information:**

The online version contains supplementary material available at 10.1007/s00068-026-03098-4.

## Introduction

The calcaneus is the largest and most frequently fractured tarsal bone. Overall, these fractures account for 2% of all fractures and are most frequently associated with high-energy trauma, such as falls from great height, which account for 71.5% of calcaneal fractures [1–4]. Consequently, they lead to significant impairment of the lower extremity function due to severe pain and progressive arthritis of the hindfoot. The incidence differs significantly between the male and female population (11.5 per 100,000 inhabitants per year versus 6.26 per 100,000 inhabitants per year, respectively). In 10% of cases, fractures of the calcaneus are bilateral [1–4].

Various treatment strategies are available for intra-articularly displaced calcaneal fractures [5–7]. According to current studies and the recommendations of the Arbeitsgemeinschaft für Osteosynthesefragen/Orthopaedic Trauma Association (AO/OTA), the current standard is the surgical treatment by means of an open reduction and subsequent internal fixation (ORIF) using a fixed-angle plate [8]. Osteosynthesis of complex calcaneus fractures using anatomically preformed fixed-angle plates, the so-called *internal-fixators*, promises the highest grade of biomechanical stability and the ability to address various fracture fragments by providing multiple options for screw placement. However, accurate placement of such plates on the calcaneal bone requires extensive surgical approaches. As a result, various minimally invasive procedures have been investigated in recent years to reduce the risk of complications [9–11]. These include the use of percutaneous screw osteosynthesis alone (without using a plate). Different types of screws and insertion techniques have been developed including cannulated screws (i.e., hollow screws that can be inserted via a guide wire), non-cannulated screws, and absorbable screws. Percutaneous screw osteosynthesis offers various advantages, such as the possibility of minimally invasive insertion with sufficient repositioning stability [12].

Previous studies and systematic reviews in recent years have described various surgical approaches, but only marginally compared the actual treatment methods [13]. Comparing single percutaneous screw osteosynthesis with ORIF is important, as this method could represent a less invasive and tissue-sparing surgery, while still achieving the best possible outcome for the injured person.

The aim of this systematic review was to examine the harms and benefits of percutaneous screw osteosynthesis for the treatment of intra-articularly displaced calcaneal fractures in comparison with ORIF.

## Methods

This systematic review is reported according to the Preferred Reporting Items for Systematic Reviews and Meta-Analyses (PRISMA) guidelines [14]. The methodological process was guided by the principles outlined in the Cochrane Collaboration Handbook [15]. The study was registered with PROSPERO (registration no: CRD42021244695). Only a short overview of the methods used in this systematic review is presented, and a more detailed version can be found in the published protocol [16].

### PICOS (Population, Intervention, Comparison, Outcomes and Study Design)

The inclusion criteria and outcome measures were conceptualized according to the PICOS Scheme. The criteria used are listed in Table 1. In deviation to the protocol only studies with the comparator intervention ORIF were eligible, as this is the most common intervention and the inclusion of different types of comparator interventions would have increased heterogeneity. In line with the Cochrane recommendation, we classified randomized controlled trial (RCT) if the authors of the included studies explicitly state that the groups compared were established by random allocation. If authors of included studies did not state this explicitly, but randomization can also not be ruled out or a quasi-randomization e.g. randomizing by date of birth was used, the studies were classified as controlled clinical trial (CCT). For further explanations please see the published protocol [16].

**Table 1:** Inclusion and exclusion criteria

	Inclusion criteria	Exclusion criteria
Population	Studies with participants with	Studies with participants with
	o uni- & bilateral intra-articular displaced calcaneus fractures	o Sanders type I (non-displaced fractures)
	o Sanders types II, III and IV or other classification system with equivalent fracture severity	o medical conditions that influence the outcome e.g., vascular and neurological diseases, diabetes mellitus and known local or systemic infections
	o Age ≥16 years	o Polytrauma
		o Severe injuries to the ipsilateral lower extremity
		o Open fractures
Intervention	Percutaneous screw osteosynthesis	Operations in which screws are only used for fracture reduction but not for fixation
Comparison	ORIF	Non-surgical treatment
Outcome	Primary outcomes:	
	o Severe complications	
	o Pain	
	o Health-related quality of life	
	Secondary outcomes:	
	o Minor complications	
	o Functional outcomes (measured by radiological parameters (if provided e.g., Bohler angle and by functional assessment instruments e.g., AOFAS-Score)	
	o Duration of surgery	
Study design	Controlled clinical trials (CCT), Quasi-Randomized controlled trials, Randomized controlled trial (RCT)	All other study designs

### Search strategy and selection process

A database search was performed using MEDLINE via PubMed, Cochrane Central Register of Controlled Trials (CENTRAL), Cumulative Index to Nursing and Allied Health Literature (CINAHL), Web of Science via Science Citation Index, and bibnet.org, and the clinical trial registries: International Clinical Trials Registry Platform and ClinicalTrials.gov. The search strategy can be found in Online Resource 1 – search strategy. The timeframe of the search was limited to all studies published after 2000, as the method of percutaneous screw osteosynthesis was first described in 2006. Furthermore, only publications published in German and English language were included. The selection process was performed by two authors (LW & AD) using Covidence software for title/abstract and full-text screening [17]. The disputes were resolved by a third author.

### Data extraction

Data were extracted independently by two authors (LW &AD) in Covidence [17]. Potential disputes between the two authors were resolved by a third author.

#### Risk of bias

Two authors independently assessed the Risk of Bias (LW & AD) in each included study using Risk of Bias Assessment Tool from Cochrane using Covidence [17, 18]. Disputes were resolved through discussion [19].

#### Data synthesis

The meta-analysis was conducted using the random-effects model where appropriate. In cases of rare events, the Mantel-Haenszel method with the fixed-effects model was used. Dichotomous data were analysed as relative risk (RR) and continuous data as mean difference (MD) or standardized mean difference (SMD) in cases where different measurement tools with the same underlying concept were used [20]. In cases of considerable heterogeneity (*I*^*2*^ > 75%), a meta-analysis was not performed. Instead, the results were described narratively.

In case of missing data, the corresponding authors of the study were contacted and asked to provide the missing data. Information regarding author contact can be found in Online Resource 2 – author contacts. A sensitivity analysis was carried out for studies with a high risk of bias and those with missing data. This analysis was also performed for the included (Quasi-) RCTs and CCTs [21]. Subgroup analysis and analysis of publication bias could not be performed [22, 23].

#### Confidence in cumulative evidence

Two authors independently (AN &DS) assessed the confidence of evidence of the primary outcomes using Grading of Recommendations, Assessment, Development and Evaluation (GRADE) [24, 25]. Disputes were solved through discussion.

## Results

**Fig. 1:**
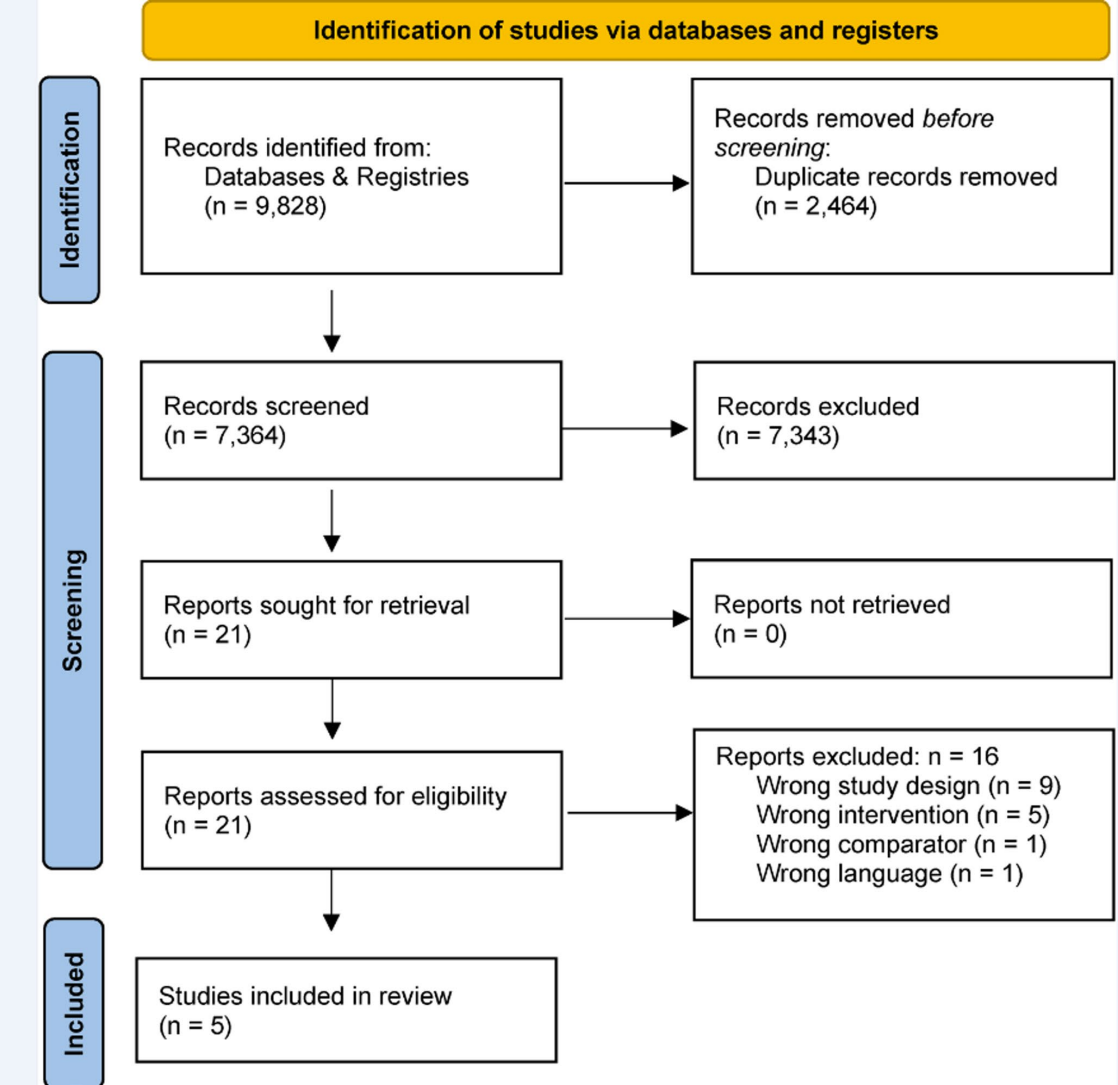
PRISMA flowchart

Five databases and two registries were searched on July 1, 2022. In total, 3,310 publications were identified in the searched databases. After excluding 885 duplicates, 2,424 publications were screened for title and abstract. 2,413 studies were excluded during this process as they did not fulfil the inclusion criteria. Of the eleven remaining, six studies were excluded. The search was last updated on January 29, 2024, with 6,518 hits (1,579 duplicates). No new study was identified. An overview of the excluded studies can be found in Online Resource 3 – excluded studies. Five studies with 6 publications were included as illustrated in Fig. 1.

### Study characteristics

Three of the included studies were RCTs [26–28] and two studies were CCTs [29, 30]. A total of 746 participants with 754 fractures were treated. One of the studies included eight patients with bilateral fractures [26]. The oldest study was published in 2011 [29], and the most recent one in 2020 [28]. Four of the five studies were from China [27–30]. The mean age of study participants was 38.4 years. 41.6% of participants were female (excluding Sampath (2014) [26], who reported sex per fracture). Fractures were classified according to the Sanders classification in all included studies. There were 337 Sanders type II, 229 type III and 188 type IV fractures as shown in Table 2. Regarding surgical techniques in the screw osteosynthesis group, all studies used a percutaneous incision (including pin und stab incisions). All studies used cannulated screws. The number of screws varied between 2 and 4 screws. In relation to the comparator intervention all studies performed an ORIF procedure with different plates (e.g. anatomical plate, calcaneus plate) and incision techniques (e.g., sinus tarsi, L-shape incision via lateral approach). Two did not state which kind of osteosynthesis was used [26, 30]. Four studies used bone grafting [26, 27, 29, 30] – two only in the screw osteosynthesis group [27, 29] and two more only in the comparator group [26, 30]. Online Resource 4 – General results of included studies provides an overview of the results of included studies.

**Table 2:** Characteristics of included studies

Study ID	Country	Study design	Sample size ^a^	Mean age ^b^	F/M	Intervention	Sanders classification		Type of incision ^c^	Osteosynthesis type ^c^	Nr of screws used	Bone grafting & type
Chen 2011	China	CCT	78	31,9	34/44	Screw fixation	II III	29 9	6.5-mm Schanz pin via stab incision	6.5- and 3.5-mm cannulated screws	4	Yes, calcium sulfate cement
						comparator	II III	32 8	extensile lateral approach	calcaneal plate		no
Feng 2016	China	RCT	80	40,1	14/66	Screw fixation	II III	32 10	6.5-mm Schanz pin via stab incision	6.5- and 3.5-mm cannulated screws	4	Yes, calcium sulfate cement
						comparator	II III	30 8	sinus tarsi approach (MISTA) front incision	anatomical plate		no
Li 2020	China	RCT	59	39,2	14/45	Screw fixation	II III IV	9 13 9	percutaneous stab incision	6.5-mm diameter hollow screw	2–3	no
						comparator	II III IV	8 11 9	L-shape incision (lateral approach)	Lateral calcaneus plate		No
Sampath 2014	India	RCT	45	31,1	n.r.	Screw fixation	II III IV	7 9 6	pin insertions	Cannulated cancellous screws (4 mm)	≥ 3	no
						comparator	II III IV	2 10 11	lateral approach with an L-shaped incision	n.r.		Yes, autologous bone graft
Wang 2015	China	CCT	492	39,7	210/282	Screw fixation	II III IV	95 76 75	percutaneous (pin insertions)	cannulated screw of 4–5 mm diameter	2–3	no
						comparator	II III IV	93 75 78	L-shape incision from Achilles’s tendon to planta pedis	n.r.		Allogeneic bone

n.r. = not reported, ^a^ number of fractures, ^b^age in years, ^c^ taken verbatim from studies

### Risk of Bias Assessment

**Fig. 2:**
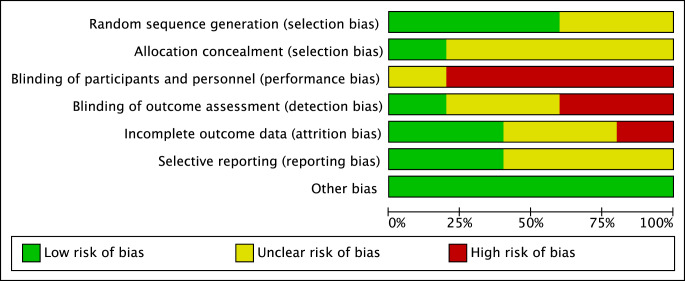
Overall Risk of Bias

The included studies generally showed a high proportion of high or unclear risk of bias mostly due to inadequate reporting for example in relation to blinding process and allocation concealment. Figure 2 illustrates the results of the individual categories of the risk of bias assessment.

**Fig. 3:**
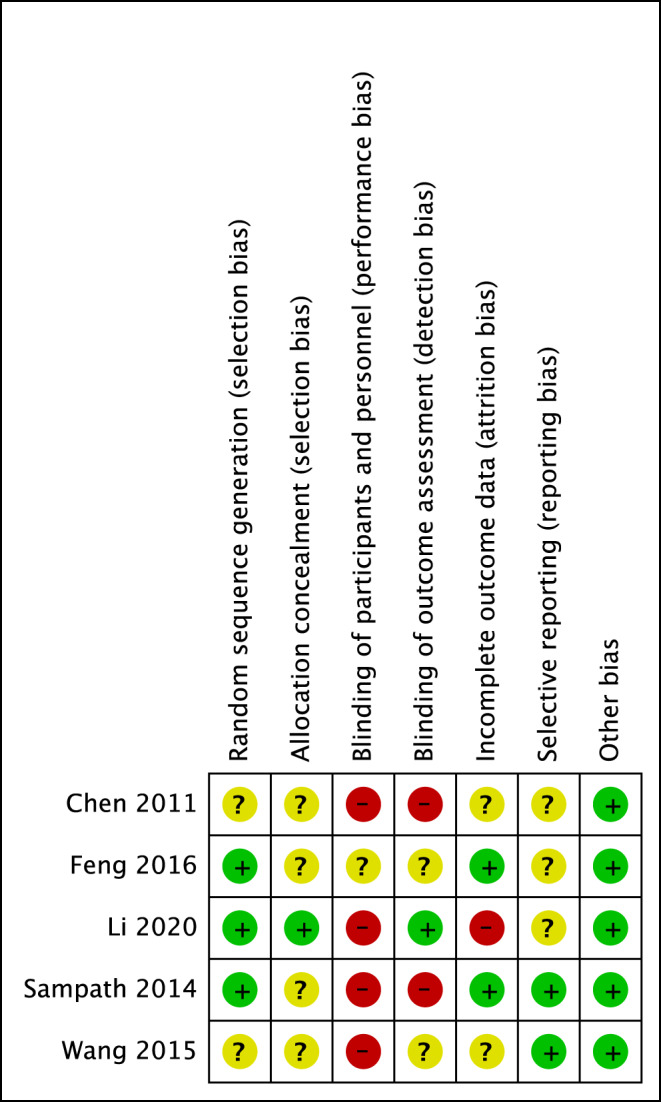
Risk of Bias of the individual studies

In two of the included studies, the risk of bias due to the generation of a random sequence remained unclear [29, 30]. All other studies described the randomization process in detail as shown in Fig. 3. All five studies showed a risk of bias in blinding of study personnel and participants, as is frequently the case in surgical studies. One study claimed that they blinded the investigating physician but did not provide any details [28]. Two of the included studies had a high risk of bias due to incomplete data [28, 29]. Li (2020) excluded twelve participants from their final analysis because these participants were followed up for less than twelve months. The authors did not specify the reasons for study participants’ dropout [28]. Similarly, Chen (2011) lost twelve study participants during the follow-up period without mentioning specific reasons for dropout.

### Results of outcome analysis

#### Severe complications

**Fig. 4:**
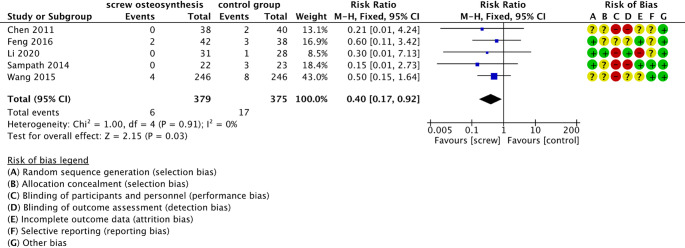
Forrest plot - severe complications

All five included studies investigated the occurrence of serious complications. Deep wound infections and traumatic arthritis were analyzed as serious complications [26–30]. Five studies with a total of 754 calcaneus fractures were included in the meta-analysis [26–30]. Figure 4 illustrates that fractures in participants who were treated using percutaneous screw osteosynthesis had a 60% lower risk of severe complications than those treated with ORIF (RR 0.40 (95% CI [0.17; 0.92]; I² = 0%; *p* = 0.03). Moderate certainty evidence shows that percutaneous screw osteosynthesis could potentially result in 27 fewer complications per 1,000 treated calcaneus fractures than ORIF (Online Resource 5).

#### Pain level

One study examined pain level using the Visual Analog Scale (VAS) (scale 1–10, higher number corresponding to greater pain) [28]. The authors found a statistically significant lower pain level in patients who underwent the percutaneous screw osteosynthesis at 24, 48, 72h, and after four weeks. For example, at 24h, they found a mean pain level of 5±0.9 in study participants treated with percutaneous screw osteosynthesis and a mean pain level of 8±0.7 (p-value = *p* < 0.001) in patients treated with the comparator intervention. After 12 weeks, the pain level did not differ significantly. However, the certainty in the evidence is low for this outcome.

#### Quality of life

None of the included studies examined the health-related quality of life (hrQoL).

#### Minor complications

**Fig. 5:**
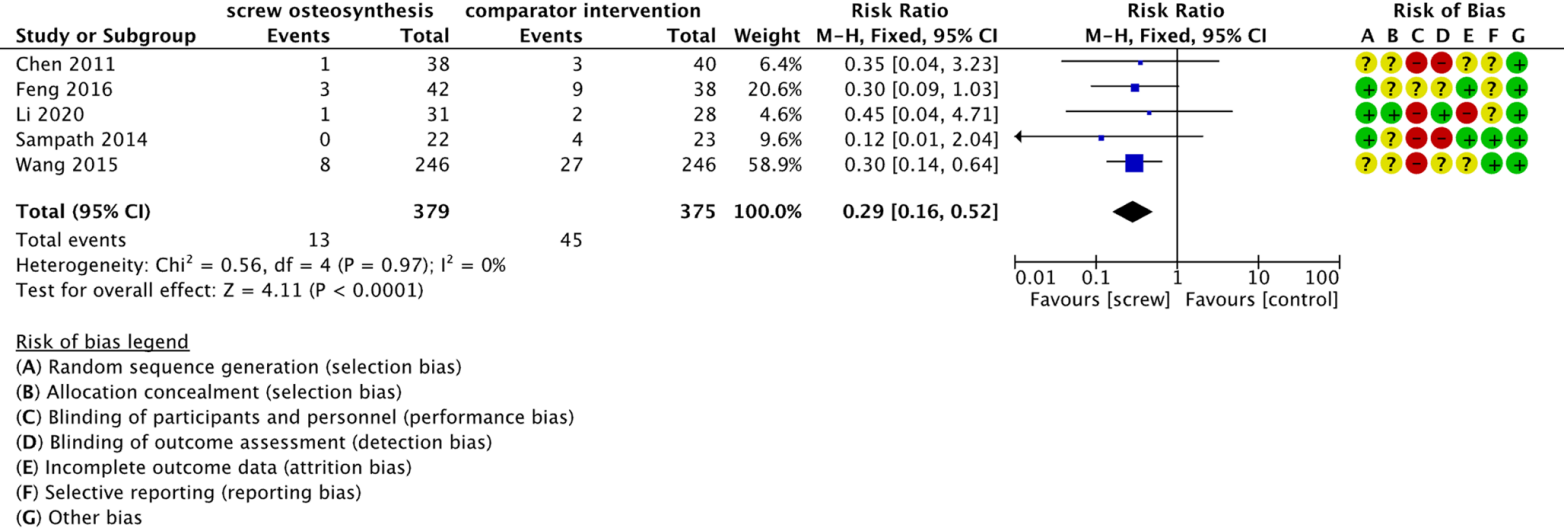
Forrest plot – minor complications

All studies included in the meta-analysis investigated the occurrence of other minor complications (e.g., superficial infections). The meta-analysis included five studies with 754 calcaneal fractures [26–30]. Fractures of participants treated with percutaneous screw osteosynthesis had a 71% lower risk of developing a minor complication than those treated with ORIF (RR 0.29; 95% CI [0.16; 0,52]; *p* < 0.001; I² = 0%), as illustrated in Fig. 5. GRADE shows moderate certainty evidence that percutaneous screw osteosynthesis could potentially lead to 89 fewer minor complications in 1,000 treated calcaneus fractures than ORIF (Online Resource 5 - GRADE Assessment).

#### Functional outcomes

**Fig. 6:**
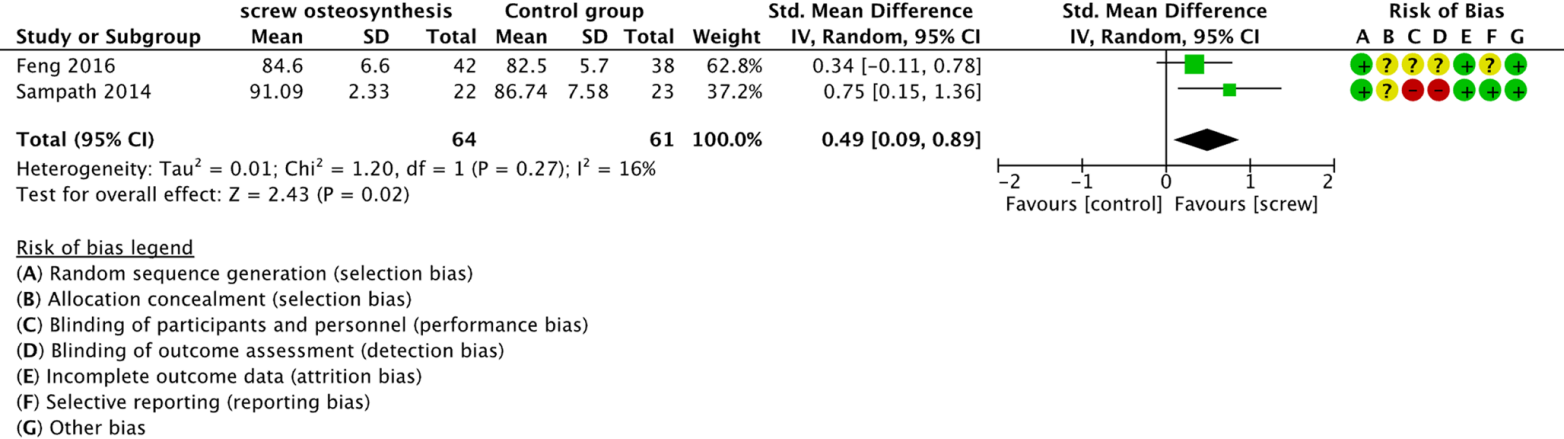
Forrest plot - Standardized mean difference of functional outcome scores

Three of the included studies [27–29] investigated pain, function, and alignment of the foot or calcaneus using the AOFAS-Score (higher number correspond with greater function), and one study used the Creighton Nebraska Health Foundation scale (CNF; higher numbers correspond with greater function) [26]. Two of the included studies did not report a standard deviation, and thus, could not be included in the meta-analysis [28, 29]. The meta-analysis of functional outcomes included two studies with 125 calcaneal fractures. Sampath 2014 used the CNF at 12 months follow-up and Feng 2016 used the AOFAS at 24 months follow-up. There were statistically significant differences in foot and gait function in favour of the percutaneous screw osteosynthesis (SMD 0.49; 95% CI [0.09; 0.89]; I² = 16%; *p* = 0.02), as shown in Fig. 6. Here, the confidence of the evidence is low (Online Resource 5 - GRADE assessment).

### Radiological indices

Statistically significant differences were found for calcaneus length directly postoperatively in favour of percutaneous screw osteosynthesis (MD -1.36; 95% CI [-2.43, -0.29]; *p* = 0.01; I^2^ = 0%; *n* = 139) [27, 28]. For most outcomes only one study reported results. No statistically significant difference was detected for all other radiological indices and measurement timepoints [27, 28]. The meta-analyses can be found in Online Resource 6.

### Duration of surgery

Three studies examined the duration of surgery [26–28]. Due to the high degree of heterogeneity (I² = 96%), no meta-analysis was performed. Two of these studies showed an average surgical duration of 39.7 and 34 min for percutaneous screw osteosynthesis, respectively [27, 28].

#### Sensitivity analysis of meta-analysis

Sensitivity analysis on the inclusion of CCTs [29, 30] and of studies with a high risk of bias depicted no influence on the stability of the results of the meta-analyses.

## Discussion

The results of the present systematic review suggest that single percutaneous screw osteosynthesis could be superior to other more invasive surgical treatments in terms of complications, postoperative pain level and function. As our analyses shows that percutaneous screw osteosynthesis for calcaneal fractures had statistically significantly fewer minor and severe complications, improved function as well as lower postoperative pain level as compared to ORIF. However, none of the individual studies showed significant differences regarding severe complications and most of the individual studies showed no difference in minor complications. Regarding the parameter “pain” only one study found a statistically significant difference in their analysis and for functional outcome only two studies were included in meta-analysis with a low certainty of evidence for this outcome.

Percutaneous, minimally invasive techniques for calcaneal fracture stabilization have the advantage of small incisions, resulting in fewer wound complications and less swelling, which can potentially cause long-term pain. These obvious advantages explain the excellent short- and medium-term results of these studies. However, the extent to which poorer fracture reduction using a minimally invasive technique will lead to a higher rate of subtalar arthrosis or arthrosis of the remaining tarsal bones in the long term remains to be seen. In principle, however, it will be difficult to distinguish between the causes of tarsal osteoarthritis development due to fracture morphology and those due to residual intra-articular step formation and malalignment of the hindfoot axis. The percutaneous technique lacks macroscopic assessment of the reduction result, and due to the often severe metaphyseal compaction of the fractures, minimally invasive techniques do not enable axial deformity and length loss to be restored to the same extent as the open technique, which is easy to control.

The included studies divided calcaneus fractures according to the Sanders classification. Significantly fewer type III fractures were included than type II fractures. The least frequent was the Sanders type IV. This can be explained by the exclusion of patients suffering a polytrauma which led to a confounding in regard to co-morbidities or co-injuries that prevent a focused assessment of the outcome of the surgical intervention in the individual studies and the present systematic review. Another possible explanation might be that due to the fracture morphology, Sanders Type IV fractures are usually not treated with percutaneous screw fixation alone as a sufficient fracture treatment. Hence, no separate conclusions can be drawn as to whether percutaneous screw osteosynthesis could also achieve good functional results for Sanders type IV fractures, as no explicit data were provided. However, it remains to be noted that the studies that included Sanders type IV fractures reported more mild and severe complications. The Sanders classification although easy to use may not adequately reflect the true fracture morphology and degree of dislocation. Although Sanders II, III, and IV subcategories count the fracture fragments affecting the subtalar joint surface, they do not allow any conclusions to be drawn regarding the degree of dislocation relative to each other or the degree of sagittal tilt and plantar compression. Hence, the classification can be misleading and misrepresent the severity of the true fracture, but other classification systems are often too complex to use in daily practice [31, 32]. Nonetheless, in times of minimal invasive approaches a classification system developed in times in which open reduction and internal fixation was the standard of care, it may not be useful for deciding whether a fracture should be treated minimally invasive or open. New classification systems are needed to reflect and aid surgeons in these decisions.

### Complications

In the meta-analysis, the risk to develop a severe complication was 60% lower and for a minor complication 71% lower for calcaneus fractures treated with percutaneous screw osteosynthesis when compared to ORIF. However, the results should be viewed with caution as none of the individual studies could demonstrate statistically significant results regarding severe complications, probably due to the combination of small sample sizes and rare events. The statistical significance of the pooled result is highly sensitive to the event rates of the individual, underpowered studies. The point estimate should therefore be interpreted with caution, as it may not be robust. The low number of observed severe complications in the present systematic review is most likely also a result of the strict distinction between severe and minor complications. The systematic review by Wang (2021) showed statistically significant results in favour of the percutaneous screw osteosynthesis. Wang (2021) included two more studies which did not fulfil the inclusion criteria for the present systematic review [33–35]. In other studies that investigated minimally invasive surgical methods, severe complications were also observed less frequently than in open surgical interventions [36–38]. Other work from the field of calcaneal fractures also found significantly less complications when minimally invasive procedures were used such as the systematic review by Marouby (2020) who examined percutaneous arthroscopically assisted calcaneus surgery [39]. They demonstrated a very low rate of wound complications and concluded that the short length of incisions is associated with a reduced rate of wound infections [39].

Accordingly, minimally invasive percutaneous screw osteosynthesis pose a lower risk of wound-related complications than other more invasive surgical interventions. Thus, this does not seem to be exclusively due to the percutaneous screw osteosynthesis, but a general phenomenon due to a more soft-tissue-sparing, minimally invasive incision technique. Screws which are inserted via a percutaneous approach have often a comparably worse reposition quality as an open approach as this allows for better fracture inspection and reduction control during surgery. Hence, percutaneously inserted screws may also have a higher risk of osteoarthritis in long-term. At least, this is the impression of experienced foot surgeons, who have observed that fracture reduction and control of the reduction of the subtalar joint surface is significantly more limited with the minimally invasive percutaneous technique. To understand this heterogeneity in surgical techniques and its impact especially on the long-term function and complications, comparative studies are needed.

#### Pain level

The data suggest that it is possible that postoperative pain may be reduced in patients with the percutaneous screw osteosynthesis but another systematic reviews in the field could not demonstrate any statistically significant difference. Peng (2021) compared open surgical procedures and the minimally invasive sinus tarsi approach for the treatment of intra-articular displaced calcaneus fractures. They were unable to detect statistically significant differences between intervention groups [40]. Also, other studies from the field of extended foot surgery, too, did not find statistically significant differences between open surgical and minimally invasive methods with regard to the pain like the systematic review by Alimy (2023) who compared the two interventions in the area of forefoot surgery [41].

#### Health-related quality of life

HrQoL was not examined in any of the included studies even though this outcome is becoming increasingly important [42]. Hence, it remains unclear which intervention leads to better hrQoL outcomes.

### Functional outcomes

Our results showed statistically significant difference between percutaneous screw osteosynthesis and ORIF. The SMD of 0.49 indicates a noticeable effect for better function in those treated with a percutaneous screw osteosynthesis. However, the certainty in evidence of these results is low amongst others due to the small sample size of 61 and 64 participants in intervention and control groups, respectively. Other systematic reviews found no statistically significant differences when examining functionality using the AOFAS and Maryland Foot Scores. However, the results cannot be directly compared due to high heterogeneity in Wang (2021) [35].

The most commonly used measurement instrument is the AOFAS, even though this instrument has come under increasing criticism for its issues with reliability, sensitivity and lack of objectifiable functional parameters. Most studies achieved above-average AOFAS scores without further explanation or qualification of the significance of these scores in measuring the restoration of function in patients with calcaneus fractures. Furthermore, scores like AOFAS and others are only inadequately able to reflect on functional outcomes in relation to differences in surgical techniques as short-term benefits in e.g. percutaneous screw osteosynthesis may be replaced by long-term consequences such as osteoarthritis. Future studies should explore more patient-reported outcomes measures specific to foot and ankle research such as the Foot and Ankle Ability Measure (FAAM) ideally in combination with a global hrQoL tool such as the SF-12 or EQ-5D [43–46].

### Operation duration

Due to high heterogeneity, a meta-analysis was not performed. The large heterogeneity in surgical duration partly resulted from different surgical care strategies. However, three of the four studies showed a shortened duration of surgery in the percutaneous screw osteosynthesis group, which is why this method could mark a faster treatment and, thus, a reduced anaesthesia risk in the future. In two of the included studies, percutaneous screw osteosynthesis took approximately 20 min less time than ORIF [27, 28]. Hence, percutaneous screw osteosynthesis may offer the possibility of a shortened surgical duration in the future. The extent to which the possible time-saving depends on the surgical intervention itself should be clarified in further RCTs.

### Limitations

Missing data on characteristics of study participants and results were a common issue despite the several attempts of contacting the corresponding authors of the included studies. Due to the small study population and the limited variability of the characteristics of the included population, the results cannot be directly transferred to the general population. Even though, the included studies reflect the average calcaneus fracture patient with an average age of 37.8 years, a sex distribution with disproportionally more males and the expected fracture type distribution [47]. However, when compared with other systematic reviews that addressed minimally invasive surgical procedures for intra-articular displaced calcaneal fractures, similar results were observed, particularly regarding mild and severe complications. Owing to the inclusion of fewer than ten studies, publication bias could not be analysed. However, various measures were taken to counteract the potential publication bias, as described in the Method section.

## Conclusion

The present systematic review indicates that minimally invasive percutaneous screw osteosynthesis for the treatment of intra-articularly displaced calcaneal fractures could result in fewer minor and severe complications as well as less pain. Further, for the long-term outcome (< 12 months) a noticeable effect of better function for the percutaneous screw osteosynthesis was found. However, our certainty in the evidence and instruments used to measure function are low. HrQoL was not investigated by any of the included studies and offers the potential for future RCTs to investigate the relevance of percutaneous screw osteosynthesis for these patients. Future studies with larger sample sizes are needed to confirm the results of this systematic review and to investigate the use of percutaneous screw osteosynthesis for different patient populations and its impact on long-term complications.

## Supplementary Information

Below is the link to the electronic supplementary material.

Supplementary Material 1

Supplementary Material 2

Supplementary Material 3

Supplementary Material 4

Supplementary Material 5

Supplementary Material 6

## Data Availability

All data generated or analysed during this study are included in this published article and its supplemental information files.

## Abbreviations

AOFAS: American Foot and ankle society
AO: Arbeitsgemeinschaft für Osteosynthesefragen
CCT: controlled clinical trials
CI: confidence interval
CNF: Creighton Nebraska Health Foundation Scale
EQ-5D: European Quality of Life 5 Dimensions 3 Level Version
GRADE: Grading of Recommendations, Assessment, Development and Evaluation
HrQoL: health-related quality of life
MFS: Maryland Foot Score
MD: mean difference
N: sample size
ORIF: open reduction and internal fixation
PRISMA: Preferred Reporting Items for Systematic Reviews and Meta-Analyses
RCT: randomized clinical trials
RR: relative risk
SMD: standardized mean difference
SF-36: Short Form 36
PSO: percutaneous screw osteosynthesis
VAS: visual analog scale
