# Supplementary material for: Percutaneous screw osteosynthesis for the treatment of intra-articular displaced calcaneus fractures
Source: Eur J Trauma Emerg Surg. 2026 Apr 21;52(1):141. doi: 10.1007/s00068-026-03098-4 (PMC13099796; doi:10.1007/s00068-026-03098-4)
Supplement: Supplementary file 1 — Supplementary Material 1 [file 68_2026_3098_MOESM1_ESM.docx]

# Online Resource 1 – search strategy

**Suchstrategien - Cochrane Library:**

#1 MeSH descriptor: [Calcaneus] explode all trees

#2 (calcan* OR calsis)

#3 #1 or #2

#4 MeSH descriptor: [Surgical Procedures, Operative] explode all trees

#5 (operative surgical procedures)

#6 MeSH descriptor: [Bone Screws] explode all trees

#7 MeSH descriptor: [Bone Plates] explode all trees

#8 (screw*)

#9 ("percutaneous")

#10 ("minimally" AND "invasive")

#11 #4 or #5 or #6 or #7 or #8 or #9 or #10

#12 #3 AND #11

**Suchstrategie - CINAHL**

(((((Calcan* OR (Calcaneus/injuries [Mesh] OR “Calcaneus fracture” [Title/Abstract] OR “calcaneal frature” [Title/Abstract]) AND (S2) AND “Screw fixation” [All fields] OR “percutaneous fixation” OR “minimally invasive" OR “calcium sulfate cement” OR “Bone Screws”) AND (S4) AND (MH randomized controlled trials OR MH double-blind studies OR MH single-blind studies OR MH random assignment OR MH pretest-posttest design OR MH cluster sample OR TI (randomised OR randomized) OR AB (random*) OR TI (trial) OR (MH (sample size) AND AB (assigned OR allocated OR control)) OR MH (placebos) OR PT (randomized controlled trial) OR AB (control W5 group) OR MH (crossover design) OR MH (comparative studies) OR AB (cluster W3 RCT)) NOT ((MH animals+ OR MH animal studies OR TI (animal model*)) NOT MH human)) AND (S8) NOT Animals OR Humans) AND (S9) ) NOT ( NOT ((MH animals+ OR MH animal studies OR TI (animal model*) NOT MH human) )

**Suchstrategie – MEDLINE via PubMed:**

1. ((((("Calcaneus"[Mesh]) OR ("Calcaneus/injuries"[Mesh] OR "Calcaneus/surgery"[Mesh] ) )) OR ("calcaneus fracture"[Title/Abstract])) OR ("calcaneal fracture"[Title/Abstract]))

2. ("screw fixation"[All Fields] OR "percutaneous fixation"[All Fields] OR "minimally invasive"[All Fields] OR "calcium sulfate cement"[All Fields] OR "Bone Screws"[MeSH Terms])

3. (((((((randomized controlled trial[Publication Type]) OR (controlled clinical trial[Publication Type])) OR (randomized[Title/Abstract])) OR (placebo[Title/Abstract])) OR (drug therapy[MeSH Subheading])) OR (randomly[Title/Abstract])) OR (trial[Title/Abstract])) OR (groups[Title/ Abstract]) NOT (("animals"[MeSH Terms] NOT "humans"[MeSH Terms]))

4. #1 AND #2 AND #3

**Suchstrategie - Web of Science:**

TI = (calcan* AND screw*) or AB = (calcan* AND screw*)

**Suchstrategie – bibnet.org:**

Alle Felder: Calcan* OR Kalkan* AND screw* OR Schraube OR Schraubenosteosynthese

**Suchstrategie – ICTRP:**

Calcaneus

**Suchstrategie – Clinicaltrials.gov:**

Calcaneus
