# Supplementary material for: Percutaneous screw osteosynthesis for the treatment of intra-articular displaced calcaneus fractures
Source: Eur J Trauma Emerg Surg. 2026 Apr 21;52(1):141. doi: 10.1007/s00068-026-03098-4 (PMC13099796; doi:10.1007/s00068-026-03098-4)
Supplement: Supplementary file 2 — Supplementary Material 2 [file 68_2026_3098_MOESM2_ESM.docx]

# Online Resource 3 – excluded studies

| **Study ID** | **Title / DOI** | **Reason for exclusion** |
| --- | --- | --- |
| Amani 2018 | Comparison of calcaneus joint internal and external fractures in open surgery and minimal invasive methods in patients  DOI: [10.4081/ejtm.2018.7352](https://dx.doi.org/10.4081/ejtm.2018.7352) | Wrong intervention |
| Cao 2022 | Percutaneous Cannulated Screw Fixation vs. Plating With Minimally Invasive Longitudinal Approach After Closed Reduction for Intra-Articular Tongue-Type Calcaneal Fractures  DOI: [10.3389/fsurg.2022.854210](https://doi.org/10.3389%2Ffsurg.2022.854210) | Wrong study design |
| El-Azab 2022 | A prospective comparative study between percutaneous cannulated screws and Kirschner wires in treatment of displaced intra-articular calcaneal fractures.  DOI: [10.1007/s00264-022-05521-y](https://dx.doi.org/10.1007/s00264-022-05521-y) | Wrong intervention |
| Fan 2016 | Cannulated screw fixation and plate fixation for displaced intra-articular calcaneus fracture  DOI: <https://doi.org/10.1016/j.ijsu.2016.08.234> | Wrong study design |
| Kir 2018 | Mini-plate fixation via sinus tarsi approach is superior to cannulated screw in intra-articular calcaneal fractures  Doi: 10.1177/2309499018792742 | Wrong intervention |
| Mansur 2021 | Clinical trial registration:  Bio-Integrative Versus Metallic Screws for Calcaneus Osteotomies  Registration number: NCT05018130 | Wrong comparator |
| Peng 2019 | Reduction and functional outcome of open reduction plate fixation versus minimally invasive reduction with percutaneous screw fixation for displaced calcaneus fracture  DOI: https://doi.org/10.1186/s13018-019-1162-5 | Wrong study design |
| Rastegar 2021 | Extensile approach versus minimally invasive technique in management of calcaneus fractures.  ISSN:2160-2026/IJBT0123875 | Wrong intervention |
| Sato 2022 | Comparison of screw versus locking plate fixation via sinus tarsi approach for displaced intra-articular calcaneal fractures.  DOI: <https://doi.org/10.1016/j.fas.2022.11.002> | Wrong study design |
| Sugimoto 2021 | Plate fixation through the lateral extensile approach versus cannulated screw fixation through the sinus tarsi approach for calcaneal fracture  DOI: https://doi.org/10.1007/s00590-021-03115-9 | Wrong study design |
| Wang 2021 | Cannulated screw fixation versus plate fixation in treating displaced intra-articular calcaneus fractures  DOI: https://doi.org/10.1007/s00264-021-05141-y | Wrong study design |
| Xu 2020  Xu 2021 | Clinical trial registration:  Sinus tarsi approach combined with percutaneous medial reduction by leverage technique for intra-articular calcaneal fractures  Registration number: ChiCTR2000038023  Sinus Tarsi Approach With Percutaneous Screw Fixation for Intra-Articular Calcaneal Fractures.  DOI: <https://doi.org/10.1053/j.jfas.2021.11.018> | Wrong study design |
| Zhang 2012 | A comparison of absorbable screws and metallic plates in treating calcaneal fractures: A prospective randomized trial  DOI: <https://journals.lww.com/01586154-201202000-00057> | Wrong intervention |
| Zhai 2021 | Application Comparison of Closed Reduction with Hollow Screw Internal Fixation and Open Reduction with Special Shaped Plate Internal Fixation in Calcaneal Fracture.  DOI: [10.36468/pharmaceutical-sciences.spl.231](https://dx.doi.org/10.36468/pharmaceutical-sciences.spl.231) | Wrong study design |
| Zhou 2022 | Observation on the effect of minimally invasive reduction and fixation of Sanders types Ⅱ and Ⅲ intra-articular calcaneal fractures under full arthroscopy  DOI: [10.3760/cma.j.cn101202-20220126-00028](https://dx.doi.org/10.3760/cma.j.cn101202-20220126-00028) | Wrong language |
