# Supplementary material for: Percutaneous screw osteosynthesis for the treatment of intra-articular displaced calcaneus fractures
Source: Eur J Trauma Emerg Surg. 2026 Apr 21;52(1):141. doi: 10.1007/s00068-026-03098-4 (PMC13099796; doi:10.1007/s00068-026-03098-4)
Supplement: Supplementary file 3 — Supplementary Material 3 [file 68_2026_3098_MOESM3_ESM.docx]

# Online Resource 4 - General results of included studies

Table a: General overview of results from the individual studies

| **Study ID** | **Duration of surgery** | | **Pain level** | | **Total number of complications** | | **Minor complications** | | **Severe complications** | |
| --- | --- | --- | --- | --- | --- | --- | --- | --- | --- | --- |
|  | **Intervention** | **Control** | **Intervention** | **Control** | **Intervention** | **Control** | **Intervention** | **Control** | **Intervention** | **Control** |
| **Chen 2011** | - | - | - | - | 1/38 (2.6%) | 5/40 (12.5%) | 1/38 (2.6%) | 3/40 (7.5%) | 0/38 (0%) | 2/40 (5%) |
| **Feng 2016** | 39.7 (± 7.6) | 64,2 (± 8.6) | - | - | 3/42 (7.1%) | 11/38 (28.9%) | 3/42 (7.1%) | 9/38 (23.7%) | 0/42 (0%) | 2/38 (5.3%) |
| **Li 2020** | 34 (± 3.1) | 60 (± 7.6) | 38.7 (± 27) | 39.3 (± 3.1) | 1/31 (3.2%) | 3/28 (10.7%) | 1/31 (3.2%) | 2/28 (7.1%) | 0/31 (0%) | 1/28 (3.6%) |
| **Sampath 2014** | 98.4 (± 22.8) | 75.6 (± 25.2) | - | - | 0/22 (0%) | 7/23 (43.5%) | 0/22 (0%) | 4/23 (17.4%) | 0/22 (0%) | 3/23 13.0%) |
| **Wang 2015** | - | - | - | - | 12/246 (4.9%) | 35/246 (14.2%) | 8/246 (3.2%) | 27/246 (11%) | 4/246 (1.6%) | 8/246 3.2%) |

Legend: ± - Standard deviation

Table b: General overview of results from the individual studies – functional outcomes at last follow-up

| **Study ID** | **AOFAS** | | **FFI** | | **MFS** | | **CFSS** | | **CNF** | |
| --- | --- | --- | --- | --- | --- | --- | --- | --- | --- | --- |
|  | **I** | **C** | **I** | **C** | **I** | **C** | **I** | **C** | **I** | **C** |
| **Chen 2011** | 91.7 (Ø) | 85.8 (Ø) | - | - | 91.5 (Ø) | 86 (Ø) | - | - | - | - |
| **Feng 2016** | 84.6 (± 6.6) | 82.5 (± 5.7) | - | - | - | - | - | - | - | - |
| **Li 2020** | 88.3 (Ø) | 86.4 (Ø) | - | - | - | - | - | - | - | - |
| **Sampath 2014** | - | - | - | - | - | - | - | - | 91 (± 2.3) | 86.7 (± 7.6) |
| **Wang 2015** | - | - | - | - | - | - | - | - | - | - |

Legend: AOFAS - American Orthopedic Foot and Ankle Society; MFS - Maryland Foot Score; FFI - Foot function Index; CFSS - Calcaneal fracture scoring system; CNF - Creighton Nebraska Health Foundation; ± - Standard deviation; I - Intervention; C - Control; Ø - Not reported

Table c: General overview of results from the individual studies – radiological indices

| **Study ID** | **Böhler angle** | | | | **Gissane angle** | | | | **Calcaneus width** | | | | **Calcaneus height** | | | | **Calcaneus lengths** | | | |
| --- | --- | --- | --- | --- | --- | --- | --- | --- | --- | --- | --- | --- | --- | --- | --- | --- | --- | --- | --- | --- |
|  | **Post-operative** | | **Last**  **follow up** | | **Post-**  **operative** | | **Last**  **follow up** | | **Post-**  **operative** | | **Last**  **follow up** | | **Post-operative** | | **Last**  **follow up** | | **Post-operative** | | **Last**  **follow up** | |
|  | **I** | **C** | **I** | **C** | **I** | **C** | **I** | **C** | **I** | **C** | **I** | **C** | **I** | **C** | **I** | **C** | **I** | **C** | **I** | **C** |
| **Chen 2011** | 32.1  ± Ø | 30.6  ± Ø | - | - | - | - | - | - | - | - | - | - | - | - | - | - | - | - | - | - |
| **Feng 2016** | 30.3  ± 4 | 30.4 ± 3.3 | 28.6 ± 3.6 | 29.2  ± 3.5 | 120.2  ± 7.2 | 119.9  ± 6 | 117.1 ± 6.8 | 117.6 ± 6.1 | 34.7  ± 2.2 | 33  ± 1.8 | 35.3 ± 2.4 | 33.4 ± 1.9 | 40.3 ± 5.4 | 40.6 ± 4.6 | 38.7 ± 2.7 | 39.3 ± 3.1 | 67.9 ± 4.3 | 69.1 ± 2.7 | 66.9 ± 3.9 | 68.2 ± 2.6 |
| **Li 2020** | 30.2  ± 2.1 | 30,7 ±1.2 | - | - | 134.2  ± 3.5 | 132.9 ± 1.9 | - | - | 42.9  ± 1.9 | 42.5 ± 1.5 | - | - | 45.2 ± 2 | 44.6 ± 1.8 | - | - | 79.2 ± 3.4 | 80.7  ± 2.3 | - | - |
| **Sampath 2014** | - | - | - | - | - | - | - | - | - | - | - | - | - | - | - | - | - | - | - | - |
| **Wang 2015** | - | - | - | - | - | - | - | - | - | - | - | - | - | - | - | - | - | - | - | - |

Legend: ± - Standard deviation; I - Intervention; C - Control; Ø - Not reported
