# Supplementary material for: Percutaneous screw osteosynthesis for the treatment of intra-articular displaced calcaneus fractures
Source: Eur J Trauma Emerg Surg. 2026 Apr 21;52(1):141. doi: 10.1007/s00068-026-03098-4 (PMC13099796; doi:10.1007/s00068-026-03098-4)

Additional file 6 – Meta-analysis of radiological indices

Böhler angle – immediately postoperative


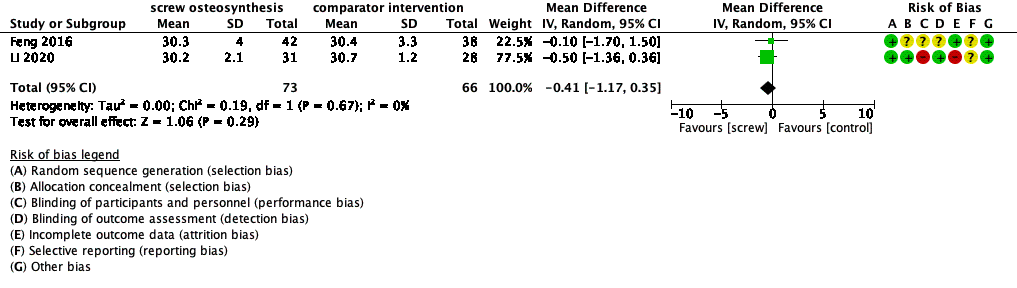


Böhler angle- last follow up


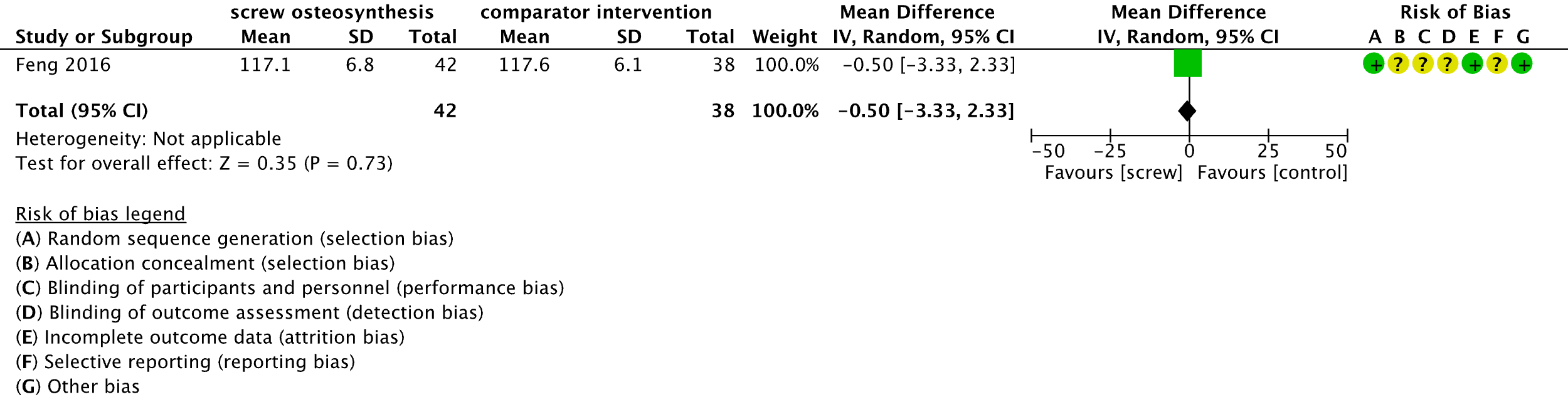


Gissane immediately postoperative


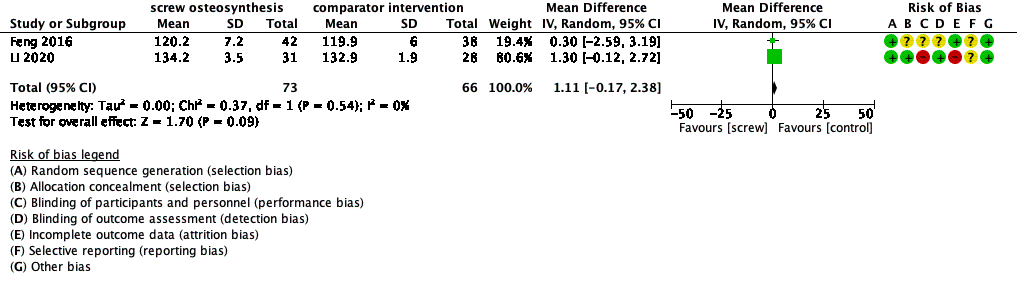


Gissane last follow up


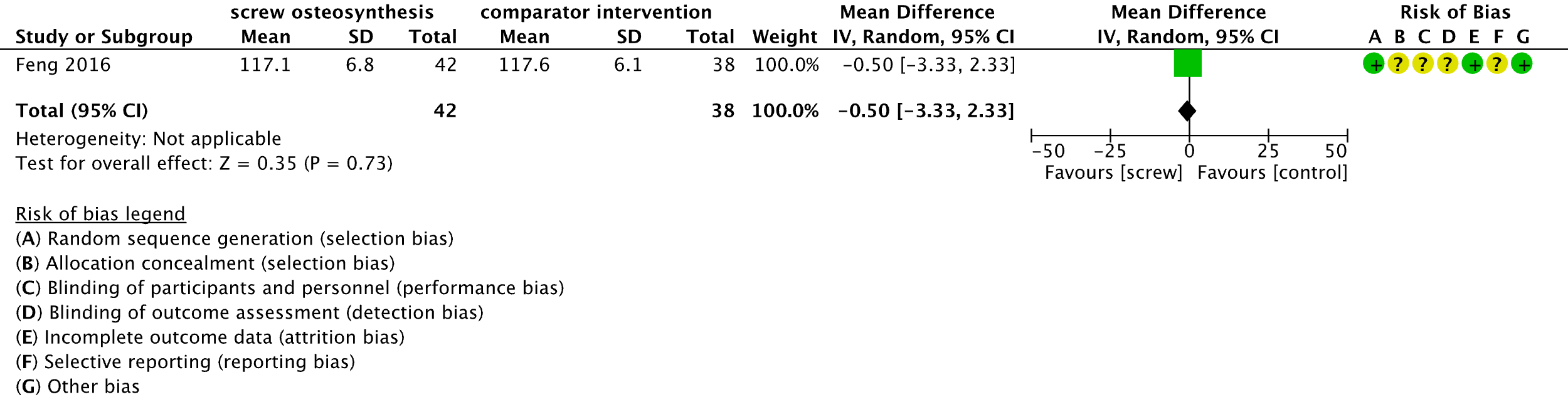


Calcaneus width – immediately postoperative


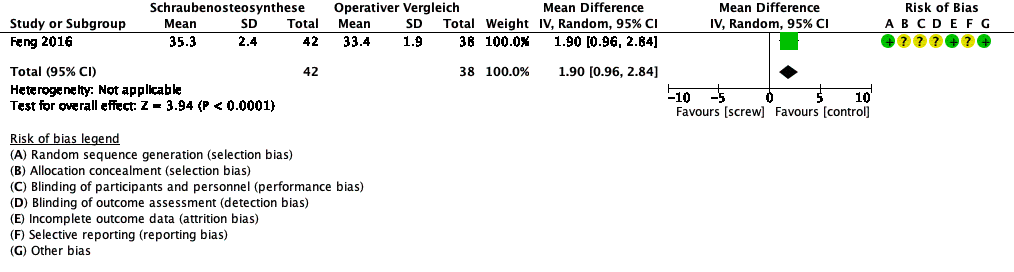


Calcaneus width – Last follow up


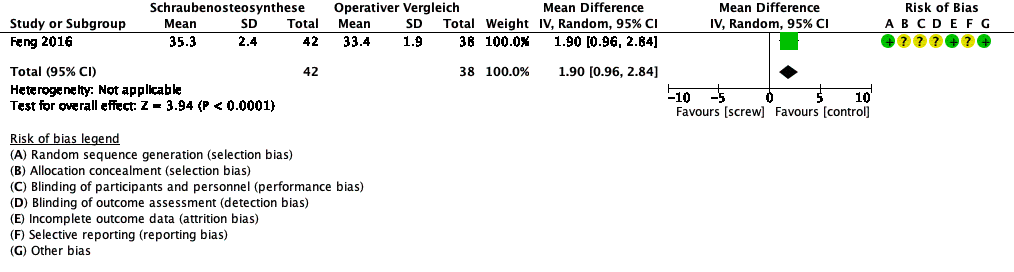


Calcaneus length – immediately postoperative


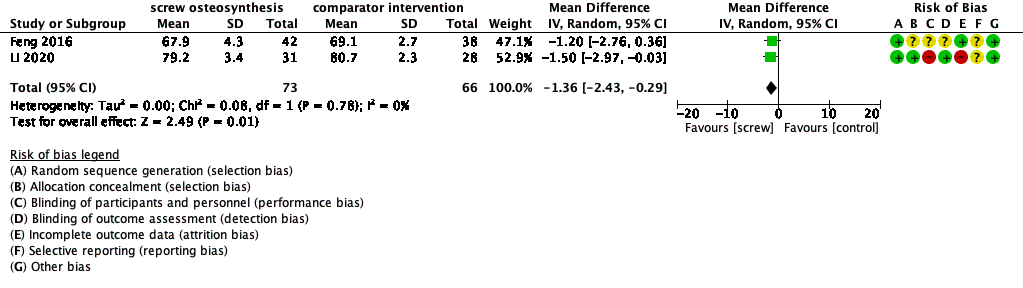


Calcaneus length – Last follow up


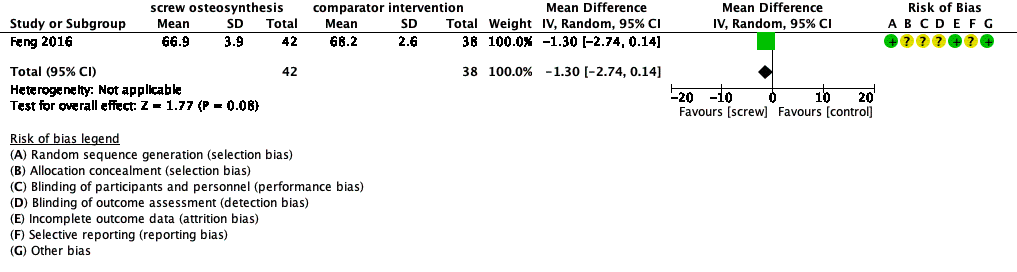


Calcaneus height– immediately postoperative


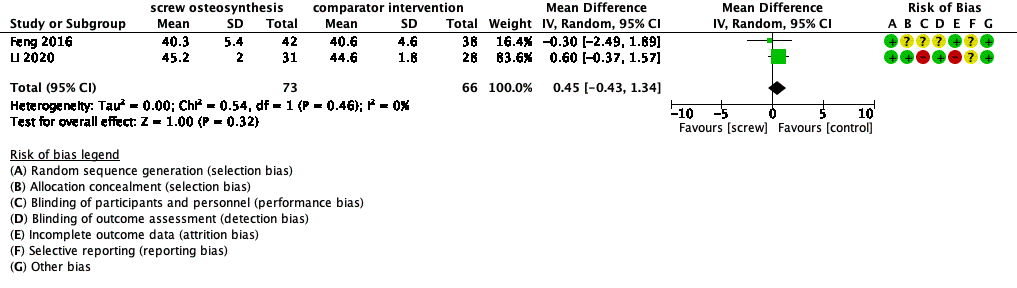


Calcaneus height – Last follow up


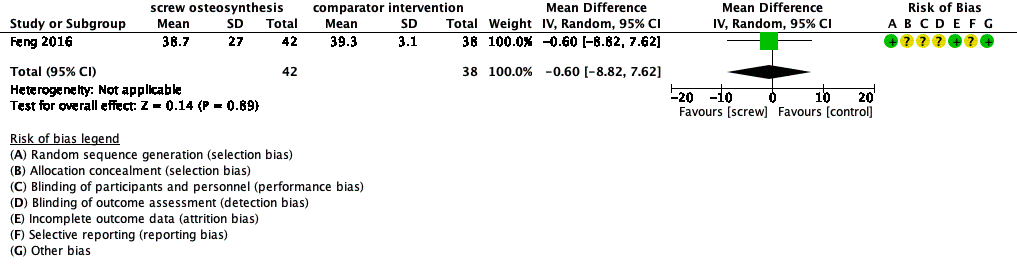

Supplement: Supplementary file 4 — Supplementary Material 4 [file 68_2026_3098_MOESM4_ESM.docx]
