# Supplementary material for: Percutaneous screw osteosynthesis for the treatment of intra-articular displaced calcaneus fractures
Source: Eur J Trauma Emerg Surg. 2026 Apr 21;52(1):141. doi: 10.1007/s00068-026-03098-4 (PMC13099796; doi:10.1007/s00068-026-03098-4)
Supplement: Supplementary file 5 — Supplementary Material 5 [file 68_2026_3098_MOESM5_ESM.docx]

# Online Resource 2 – author contact

| **Study ID** | **Corresponding author** | **Date of 1. contact** | **Inquiry** | **Response** |
| --- | --- | --- | --- | --- |
| Feng 2016 | hongjianjun_fey@163.com Department of Orthopaedics Surgery, The Second Affiliated Hospital of Wenzhou Medical University, NO. 109, Xue Yuan West Road, Lucheng District, Wenzhou, Zhejiang Province 325027, China | 13.08.2022 | Missing Data | No response |
| Li et al., 2020 | Lijie Ma, e-mail: drlm0216@126.com, Wei Chen, e-mail: drchenwei1@163.com | 14.08.2022 | Missing Data | No response |
| Chen et al., 2011 | Wen Yuan MD, Department of Orthopedics, Chang Zheng Hospital, Second Military Medical University, Feng Yang Road, 415 Shanghai, 200003 China • surgeonyuanwen@126.com | 14.08.2022 | Missing Data | No response |
| Sampath et al., 2014 | V. Sampath Kumar (*): V. Sharma: P. Kotwal All India Institute of Medical Sciences, Orthopaedics, New Delhi, India e-mail: venkatortho4@gmail.com | 14.08.2022 | Missing Data | No response |
| Li et al., 2020 | Lijie Ma, e-mail: drlm0216@126.com, Wei Chen, e-mail: drchenwei1@163.com | 07.03.2022 | Missing data | No response |
| Wang et al., 2015 | Q. Wang Á X. Li Á Y. Sun Á L. Yan Á C. Xiong Á J. Wang (&) Department of Orthopaedics, Subei People’s Hospital, 98 West Nantong Road, Yangzhou 225001, China e-mail: drjingchengwang@126.com | 07.03.2022 | Missing data | No response |
| Chen et al., 2011 | Wen Yuan MD, Department of Orthopedics, Chang Zheng Hospital, Second Military Medical University, Feng Yang Road, 415 Shanghai, 200003 China • surgeonyuanwen@126.com | 21.01.2022 | No response | No response |
| Li et al., 2020 | Lijie Ma, e-mail: drlm0216@126.com, Wei Chen, e-mail: drchenwei1@163.com | 21.01.2022 | No response | No response |
