# Supplementary material for: Percutaneous screw osteosynthesis for the treatment of intra-articular displaced calcaneus fractures
Source: Eur J Trauma Emerg Surg. 2026 Apr 21;52(1):141. doi: 10.1007/s00068-026-03098-4 (PMC13099796; doi:10.1007/s00068-026-03098-4)
Supplement: Supplementary file 6 — Supplementary Material 6 [file 68_2026_3098_MOESM6_ESM.docx]

# Online Resource 5 – Summary of findings table

| **Screw osteosynthesis compared with other surgical intervention of intraarticular dislocated calcaneus fracture** | | | | | |
| --- | --- | --- | --- | --- | --- |
| **Population:** intraarticular dislocated calcaneus fracture  **Setting:** hospital  **intervention:** screw osteosynthesis  **comparator:** other surgical interventions | | | | | |
| **Outcome** | **№ of participants (nr. study)** | **Certainty of the evidence (GRADE)** | **Relative effect (95% CI)** | **Assumed absolute risks* (95% CI)** | |
|  |  |  |  | **Risk with comparator intervention** | **Risk difference with screw osteosynthesis** |
| severe complications | 754 (5 RCTs) | ⨁⨁⨁◯ Moderate^a^ | **RR 0.40** (0.17 to 0.92) | 72 pro 1.000 | **43 less per 1.000** (60 less to 6 less) |
| pain | 59 (1 RCTs) | ⨁⨁◯◯ Low^a,b^ | - | Mean pain was **5** points | MD **0.3 less** (0.54 less to 0.06 less) |
| minor complications | 754 (5 RCTs) | ⨁⨁⨁◯ Moderate^a^ | **RR 0.29** (0.16 to 0.52) | 155 pro 1.000 | **110 less per 1.000** (130 less to 74 less) |
| functional outcome | 124 (2 RCTs) | ⨁⨁◯◯ Low^a,b^ | - | - | SMD **0.49 SD higher** (0.09 higher to 0.89 higher) |
| ***The risk in the intervention group** (and the 95% confidence interval) is based on the presumed risk in the comparison group and the **relative effect** of the intervention (and the 95% CI). **CI:** confidence interval; **MD:** mean difference; **RR:** risk ratio; **SMD:** standardized mean difference  **GRADE Working Group grades of evidence** **High certainty:** we are very confident that the true effect lies close to that of the estimate of the effect. **Moderate certainty:** we are moderately confident in the effect estimate: the true effect is likely to be close to the estimate of the effect, but there is a possibility that it is substantially different. **Low certainty:** our confidence in the effect estimate is limited: the true effect may be substantially different from the estimate of the effect. **Very low certainty:** we have very little confidence in the effect estimate: the true effect is likely to be substantially different from the estimate of effect.  **Explanation:**  a. slightly different population (unilateral vs. bilateral; Sanders Typ II, III & IV vs. II, III)  b. small sample size | | | | | |
